# Exposure the Hazardous Potential of Quinalphos: An In‐Depth Analysis for Environmental and Biological Effects on Human Health

**DOI:** 10.1049/syb2.70077

**Published:** 2026-07-28

**Authors:** Jyoti Kant Choudhari, Biju Prava Sahariah, Anand Kumar Jayapal, Jyotsna Choubey, Abhishek Tripathi

**Affiliations:** ^1^ Department of Microbiology All India Institute of Medical Sciences Bhopal Madhya Pradesh India; ^2^ National Institute of Technology Raipur Raipur Chhattisgarh India; ^3^ Raipur Institute of Technology Raipur Raipur Chhattisgarh India; ^4^ Maulana Azad National Institute of Technology Bhopal India

**Keywords:** big data, bioinformatics, biology computing, diseases

## Abstract

Quinalphos pesticide (QP) exposure can lead to various human health effects, including anaemia, leucocytosis with neutrophilia, hepatic damage and oxidative stress. Acute poisoning symptoms include weakness, sweating, impaired vision and neurological distress. Additionally, neurological and reproductive abnormalities have been reported in specific exposure scenarios. This study identified 16 differentially expressed genes from clinical exposure research. Integrating omics data using network biology approaches has shown that QP significantly alters the expression of 16 genes predicted to be regulated by 26 transcription factors and 41 miRNAs. The molecular docking predicted AR, ESR1, NR1I2, ESR2, CYP19A1 and JUN to have the highest binding affinity with QP. Gene ontology analysis of the DEGs revealed enrichment in pathways related to genital development, oestrogen receptor signalling, prostate gland development and response to vitamin A. The three most influential pathways by these DEGs are detected with significant enrichment in diseases, and they are linked to Cytochrome P450, arranged by substrate type, nuclear receptor transcription pathway and SUMOylation of intracellular receptors. The analysis identified 1445 drugs for 15 genes for potential drug repurposing. The network was pruned by applying the threshold value 0.1 STITCH database and score value 0.1 of the DGIdb database, resulting in the identification of 446 drug (approved and non‐approved) therapeutic targets. This analysis provides a comprehensive understanding of the mechanisms of QP‐induced toxicity, focusing on humans, and underscores the need for further studies on exposure to QP, providing valuable insights for toxicological risk assessment and regulatory evaluation.

## Graphical abstract

This study elucidates pesticide docking onto proteins encoded by differentially expressed genes, revealing interaction networks involving chemicals, genes, proteins, drugs and diseases. It highlights structural insights, regulatory pathways and potential therapeutic or environmental implications, providing a comprehensive understanding of the biological systems influenced by these interactions.

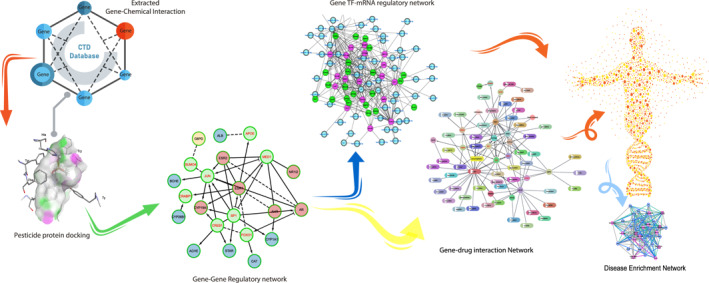

## 1 Introduction

In Indian agronomy, quinalphos (QP, O, O‐diethyl‐O‐[2‐quinoxalinylphosphorothioate]) is one of the most commonly used organophosphate pesticides for pest control to protect crops [1]. In view of a few suitable properties, for example, efficiency in quick pest control, adequate biodegradability and diminutive bioaccumulation, with the World Health Organization’s (WHO) classification as moderately hazardous and as a yellow labelled (highly toxic) pesticide in India, QP is a popular choice among farmers. The common target of QP in Indian rice, cotton and rapeseed fields are *Scirpophaga incestuous*, *Pegomya hyoscyamine*, *Lipaphis erysimi*, *Helicoverpa zea*, *Stem borers*, *Cnaphalocrosis medinalis*, *Cicadella viridis and Dicladispa armigera*. Some quinalphos formulations treat stored grains to prevent infestations by grain weevils and meal moths. QP is applied as a seed treatment to control corn rootworm and soybean aphids in field crops and as a nematicide to control poultry red mites and nematodes such as poultry lungworms. It is used as a dip for salmonids to control sea lice and several other fish parasites, including dory perch and rusty crayfish parasites. Production, transportation, and application of pesticides in the field certainly cause high exposure of the same to a significant number of humans through dermal contact, inhalation, and/or any other way [2, 3, 4, 5].

Pesticide contamination is inherently anthropogenic, arising from human practices focusing on quick profits. A realization of the problem arises from any combination of factors: population growth, economic motivations, lack of knowledge and a lack of awareness. As a result, non‐target organisms suffer the consequences of these practices and highlight the ecological costs associated with pesticide usage. The substantial purpose of a pesticide is to eliminate target species (pests and weeds). Therefore, pesticides are designed to possess toxic elements in their concentrated ability to broadly interfere with the nervous, metabolic, and reproductive systems of all pests. A common mechanism of organophosphate pesticide activity is inhibiting acetylcholinesterase (AChE) following the accumulation of acetylcholine and excessive stimulation of cholinergic neurons, and QP is no different from the above functionality [5, 6, 7, 8]. Being highly soluble in water (17.8 mg/L), quinalphos is readily transported through soils into groundwaters or surface waters. Therefore, eliminating quinalphos from the groundwater is a significant concern.

Additionally, the principal intermediates generated from photolytic degradation of QP are quinalphos oxon, 2 hydroxy quinoxalines, trialkaline phosphate and thiol metabolite, which are recognized as more toxic than parent compounds [9]. Aquatic organisms, especially freshwater fishes, are highly prone to the adverse effects of QP [3]. A decrease in serum and red blood cell cholinesterase activity is often considered an organophosphate poisoning indicator. Quinalphos acts as a neurotoxicant, inhibiting acetylcholinesterase, affecting the respiratory system and irritating the skin and eyes. Once it enters the human body, QP undergoes several biotransformation reactions, interacting with various biomolecules. Residual pesticides increase pesticide exposure folds among consumers. Comparative toxicogenomic database (CTD) recognized nearly 298 diseases associated with QP alone, including mild to various types of cancer, and diseases related to cardiovascular, blood, renal, skin, metabolic and neural systems are listed [2, 5].

The intermediates are responsible for oxidative stress, destruction of vitamin E, micronuclei formation, compensatory induction of anti‐oxidant enzymes, decrease of radical scavengers, chromosomal aberrations etc. [4, 5, 8]. QP is widely used in Indian agriculture, but investigation of the fate of QP at the cellular and molecular levels is limited. Systematic information about the QP‐influenced genes, respective regulatory factors (transcription factors), involved components, process, and function and their interaction is expected to provide suitable ways to control the impact of QPs on health and consequence knowledge to resolve the issue.

This study's primary objective is to better understand the toxic potential of QP, a commonly utilised organophosphate insecticide in agricultural pest management, gardening and veterinary applications. This study delivers a comprehensive investigation of QP interaction with people at the genetic level through network biology approaches, which goes beyond traditional toxicological assessments. The study examines alterations in the expression of specific genes, their control by transcription factors and microRNA, their correlation with diverse disorders and prospective pharmacological interventions, all of which influence human health. The research provides a systems‐level understanding of OP toxicity by mapping the alteration in gene expression, analysing their regulation by TFs and microRNAs, and correlating these changes with disease pathways and potential pharmacological intervention. Unlike previous studies that primarily document end‐point damage or biochemical shifts, this investigation constructs a detailed regulatory network that connects QP exposure to specific genetic disruption and health outcomes [10]. With omics data integration, this network biology clarifies the molecular underpinnings of QP‐induced toxicity. It identifies actionable targets for therapeutic intervention and risk mitigation, an advancement not previously achieved in OP research. It also helps to find suitable solutions to resolve the threat of QP to the biosphere.

## 2 Materials and Methodology

### 2.1 Dataset

The comparative toxicogenomic database (CTD; http://ctdbase.org/) provides high‐quality content that is contextualised better to understand the relationship between chemical exposures and human health and to assist in comprehending diseases caused by environmental factors [11]. It manually curates the scientific literature to identify chemical genes, chemical diseases, gene diseases, chemical phenotypes and chemical exposure correlations across all species. CTD users are provided with tools for analysis and visualisation that can assist them in formulating hypotheses that can subsequently be tested [12, 13].

The model's cross‐species comparative framework also supports the translational perspective from model organisms to humans. In addition, the exposure science module of CTD's system of tools constructs relevance by integrating actual molecular outcome measures, which is key for toxicological risk assessments and regulatory evaluations. Even with limitations such as bias in the literature or needing experimental support, CTD's strong, integrative data system framing environment data streams in an ontology is conducive to forming advanced theories and using design logic reasoning in environmental health research [14]. Gene interaction search features identified genes affected by exposure to quinalphos (QP). Only those genes that demonstrated a strong relationship according to the exposure studies were considered in the research. The comparative toxicogenomics database (CTD) lists 16 genes impacted by the chemical quinalphos, as shown in Table 1.

**TABLE 1:** List of genes affected by exposure to QP.

Gene symbol	ID	Interaction	Reference
ACHE	43	Quinalphos results in decreased activity of ACHE protein	[15]
AHR	196	Quinalphos binds to and results in increased activity of the AHR protein	[16]
ALB	213	[Arsenic co‐treated with quinalphos] results in decreased expression of ALB protein	[15]
AR	367	Dihydrotestosterone inhibits the reaction [quinalphos results in increased activity of AR protein]	[17]
BCHE	590	Quinalphos results in decreased activity of BCHE protein	[18]
CAT	847	Quinalphos results in decreased activity of CAT protein	[15, 19]
CYP19A1	1588	Quinalphos results in increased expression of CYP19A1 mRNA	[1]
CYP1A1	1543	Quinalphos results in decreased activity of CYP1A1 protein	[20]
CYP26B1	56603	Quinalphos results in decreased expression of CYP26B1 mRNA	[19]
ESR1	2099	Quinalphos results in increased activity of ESR1 protein	[17]
ESR2	2100	Quinalphos results in increased activity of ESR2 protein	[17]
G6PD	2539	Quinalphos affects the activity of the G6PD protein	[21]
GSR	2936	Quinalphos results in decreased activity of GSR protein	[21]
HSD3B3	15494	Quinalphos results in decreased expression of HSD3B3 mRNA	[19]
NR1I2	8856	Quinalphos binds to and results in increased activity of NR1I2 protein	[17]
STAR	6770	Quinalphos results in decreased expression of STAR protein	[19]

### 2.2 Molecular Docking

For pesticide docking, we have selected PDB IDs (4EY7, 5V0L, 1AO6, 2Q7I, 6SAM, 1DGF, 4I8V, 3S79, 1FAH, 3OS8, 1YYE, 6E08, 2GRT, 1HXH, 1ILH and 3P0L) that are coded by differentially expressed genes by the AutoDock^4.2.6^. Molecular docking uses these proteins with the quinalphos pesticide to determine the estimated free energy of binding (ΔG). The lower the energy (negative), the greater the binding affinity between the protein and the drug. The protein is prepared for docking by removal of the water molecule, addition of polar hydrogen and distribution of charge on each residue of the protein molecule. The ligand molecules are retrieved in the ‘sd’ format from PubChem and then prepared by detecting the root of the torsion tree, setting the number of torsions between l and 6. This ligand and proteins are also saved in their ‘pdbqt format’. For blind docking, the entire molecule is covered with an imaginary three‐dimensional grid box. The grid points are standard dimensionless points depending on the grid spacing value. The grid parameter file is created by selecting the appropriate ligand and macromolecule. Multiple iterations of gridding and docking are carried out with a different ligand to locate the binding site. Initially, all amino acids are used for blind docking.

### 2.3 Gene Functional Interaction Network

Gene functional interaction involves two genes in the same biochemical reaction as an input, catalyst, activator, or inhibitor. The Cytoscape ReactomeFIViz (ReactomeFI) plugin has generated and annotated a gene functional interaction network. ReactomeFIViz finds disease‐related network pathways. This plugin enables performing pathway enrichment analysis for a list of genes, visualising hit pathways using manually laid‐out pathway diagrams, and investigating functional relationships among genes in found pathways [22]. In the function interaction network analysis, 16 genes are selected based on the literature and create the functional interaction network.

### 2.4 TFs Regulatory Network

The iRegulon plugin in Cytoscape is used for the TFs Network [23]. Transcription factors (TFs) can effectively quantify gene expression regulation. The GeneSingDB curated gene signature and the MsingDB molecular signature database predict the regulatory transcript factors (TFs) for the differentially expressed genes using an iRegulon plugin for Cytoscape [24]. We have predicted regulators and targets with the help of some pre‐defined parameters.

### 2.5 Gene TF‐mRNA Regulatory Network

The manually curated data of genes is retrieved, and the interaction network uses the Reactome database. Similarly, individual networks, TF‐gene regulatory, miRNA‐TFs and gene interaction are merged into a composite multi‐layer by mapping the target node, Gene to miRNAs, TFs to genes and TFs to miRNAs, where nodes represent genes, TFs and miRNAs, and edges represent functional interactions. The CyTargetLinker plugin expands the network with two selected data link sets named ‘miRTarBase’ and ‘TargetScan.’ These databases are based on ‘microRNA‐target gene’ interaction types. To identify targets and regulators, we search for gene‐miRNA targets and cross‐validation using the microRNA data integration portal (mirDIP) in 30 database sources by applying the high integrated score (< 0.5) and more.

### 2.6 Disease Enrichment Analysis

We have used the DisGeNET plugin in Cytoscape to analyse the 16 genes identified as responsible for a disease. DisGeNET returns a list of genes associated with the investigated disease using different identifiers, namely, disease name, gene and variants [25].

### 2.7 Gene Drug Interaction Analysis

The drug–gene interaction dataset was extracted from the STITCH and DGIdb databases. These databases are specialised for analysis and retrieving the drug–gene interaction and the relationship analysis. The STITCH is a search tool and resource for the interaction of chemicals and proteins. Chemical–chemical and chemical–protein associations are integrated from the pathway, experimental databases and literature [26]. In contrast, DGIdb mainly focuses on clinically relevant drug–gene interactions. It is a valuable resource for identifying the potential therapeutic targets and supporting precision medicine efforts [27]. The STITCH web tools are used to identify 16 gene drug interactions with a minimum required interaction score of 0.150 (Low confidence). The DGIdb database is used for purging and enriching the gene‐drug interaction by applying the threshold value 0.1 (interaction score) to retrieve more information.

### 2.8 Cluster Analysis

Cytoscape's MCODE plugin [28] is used for module analysis. The analysis standards are: MCODE cutoff > 5, degree cutoff = 2, node score cutoff = 0.2, maximum depth = 100 and k‐core = 2. These parameters eliminate weakly linked or unassociated nodes, directing the focus towards densely connected clusters that are more likely to encapsulate critical biological processes and functional groups. Consequently, fourteen top‐level modules were displayed; the first four top‐level modules interacted most closely [23].

## 3 Results and Discussion

### 3.1 Quinalphos's Effect on the Genes

Quinalphos is a broad‐spectrum organophosphate insecticide widely used in households and crop fields. Epidemiology studies indicated quinalphos could affect the activity of specific proteins in the body, including AHR, AR, CYP19A1, ESR1, ESR2, NR1I2 (increased expression); ACHE, ALB, BCHE, CAT, CYP1A1, CYP26B1, GSR, HSD3B3, STAR (decreased expression) and G6PD (affects activity) genes. These genes involve various biological processes, such as metabolism, stress response and hormone regulation. Quinalphos decreases the activity of these proteins, which can disrupt their normal functions. Quinalphos inactivates acetylcholinesterase (AChE), a key enzyme for producing and storing neurotransmitters in the nervous system. Quinalphos binds to AChE's esteratic site, phosphorylating the serine residue and rendering the enzyme inactive [29]. Consequently, AChE accumulation causes muscarinic (e.g., bradycardia and miosis), nicotinic (e.g., muscle fasciculations) and central nervous system effects [30]. Losing a chemical group from the OP–enzyme complex prevents further enzyme reactivation. After this process (termed ‘ageing’) has taken place, new enzymes must be synthesised before their function can be restored [31, 32]. BChE inhibition by quinalphos is not directly evidenced in the provided studies. However, organophosphates generally inhibit both AChE and BChE, with BChE gaining relevance in advanced neurodegenerative conditions. The long‐term effects of quinalphos on these proteins are still unclear, and more research is needed to understand how quinalphos affects the body's physiology. Quinalphos exposure leads to significant hepatic (liver) damage and metabolic disturbances. In animal studies, sub‐lethal doses of quinalphos caused oxidative stress in the liver, as evidenced by decreased ferric reducing ability of plasma (FRAP), indicating reduced antioxidant capacity [33]. Quinalphos has a strong binding affinity for the aryl hydrocarbon receptor (AHR) protein, an intracellular receptor that plays a role in the body's response to environmental contaminants [16, 34, 35]. The binding of quinalphos to AHR results in increased protein activity. Recent research has shown that quinalphos can significantly affect animal ALB protein expression [36, 37]. ALB protein is a key component of the liver and plays an important role in various metabolic processes [38]. In studies involving mice exposed to quinalphos, researchers observed a marked decrease in the expression of the ALB protein, which led to several health problems. This decrease is most pronounced in the liver, where ALB is produced [39]. A recovery period can return levels of ALB to normal. Quinalphos disrupts antioxidant defence mechanisms by affecting GSR activity, impairing the glutathione recycling and detoxification capacity. Significant decreases in CAT activity are observed in multiple tissues (liver, kidney and testis) after quinalphos treatment, reducing the breakdown of hydrogen peroxide and increasing susceptibility to oxidative damage (Padmanabha et al. 2015). In a study in hamsters, quinalphos showed marked decrease in testicular antioxidant enzyme (superoxide dismutase, SOD and catalase, CAT) activities with an increase in lipid peroxidation (MDA) level. Similarly, increased AR protein expression has been reported in dihydrotestosterone (DHT), a powerful androgen hormone synthesised from testosterone, with an important role in male sexual development, and can negatively affect overall health [40]. DHT has 5–10 times higher affinity for AR than testosterone. AR‐bound DHT upregulates hair development inhibitory factors, such as DKK‐1, IL‐6 and TGF‐β, encouraging hair follicle regression [41]. Quinalphos can interfere with hormone receptors such as the androgen receptor (AR), oestrogen receptor alpha (ESR1) and oestrogen receptor beta (ESR2) [42] According to various animal studies, OP insecticides may act as endocrine disruptors. Quinalphos‐induced testicular damage and oxidative stress impair testosterone synthesis, leading to reduced testosterone levels and negatively affecting male reproductive health [43]. Additionally, disruption of the hypothalamic–pituitary–gonadal (HPG) axis by quinalphos can alter luteinizing hormone (LH) secretion, which is essential for testosterone production and spermatogenesis [44]. Serum LH levels decreased in parathion‐exposed quails but recovered to normal levels 24 h later. In rats, chlorpyrifos–methyl binds to androgen receptors and exerts anti‐androgenic effects. Sublethal doses of quinalphos increased LH, prolactin and testosterone levels in rats, whereas dimethoate lowered LH levels in sheep [45]. Butyrylcholinesterase (BCHE), another important enzyme, exhibits significant activity decreases after exposure to quinalphos [18, 46]. It is involved in the breakdown of acetylcholine, a neurotransmitter that plays an important role in the nervous system [47, 48, 49]. An experiment with a mouse model, with 2 weeks of exposure to quinalphos, specified decreased activity of the catalase CAT protein, essential for protecting cells from oxidative damage, and the findings suggest that exposure to quinalphos leads to an increased risk of oxidative damage [15, 19].

Increased activity of the ESR1 and ESR2 proteins, known to mediate the effects of oestradiol, a primary female reproductive hormone, is reported due to quinalphos exposure [50]. These results confirm that quinalphos acts as an endocrine disruptor, which can interfere with normal hormone production and signalling pathways [51]. Similarly, quinalphos can increase the expression of CYP19A1 mRNA (upregulation), an enzyme responsible for oestrogen production in humans and other mammals, increasing the production of oestrogens [1].

Quinalphos affects G6PD protein activity, the active gene encoding in red blood cells (RBCs) in either tetrameric or dimeric form [21]. The mutation type influences the level of activity of the protein in G6PD. In cases of a family history, a mutation is passed down through the family chain [52, 53]. A mutation in the gene G6PD results in a lower functional glucose‐6‐phosphate dehydrogenase, which in turn results in lower levels of the coenzyme NADPH and depletion of the anti‐oxidant glutathione, needed for the protection of the cells' haemoglobin and their cell walls (red cell membranes) from highly reactive oxygen radicals (oxidative stress). Individuals with a deficiency of G6PD cannot regenerate reduced glutathione (GSH) and are thus not protected from oxidative stress. The increased requirement of reduced equivalents by oxidative stress acting either on NADP/NADPH or the GSH system results in increased saturation of G6PD with NADP, increased intracellular G6PD activity and increased PPC flux [54, 55].

Glutathione reductase (GR) or glutathione disulfide reductase (GSR), is an enzyme encoded by the GSR gene in humans. Glutathione reductase deficiency is a rare reductase activity condition, absent in erythrocytes, leucocytes or both. The activity of glutathione reductase is used as an indicator of oxidative stress. Quinalphos affects the activity of the GSR protein by altering the activity of GSR in rat tissues [21].

Studies reveal that QP decreases testosterone levels in mice, resulting in less viable sperm. Its toxicity is caused by free radical production, indicated by increased lipid peroxidation (LPO) and decreased antioxidant enzyme levels in testicular tissue. Increased serum cholesterol and decreased testicular cholesterol levels indicate impaired transport and biosynthesis. The lack of cholesterol in testicular tissue hinders steroidogenesis by downregulating 3‐HSD, 17‐HSD and StAR, thereby reducing testosterone levels. In steroidogenic cells, hormone‐induced cholesterol transport is controlled by a protein complex containing the steroidogenic acute regulatory (StAR) protein. StAR protein functions as a key determinant of steroidogenesis via cholesterol transport from the outer mitochondrial membrane (OMM) to Cyp11a1 in the inner mitochondrial membrane (IMM) [56, 57].

Pregnane X receptor, also known as NR1I2, is a transcription factor activated by ligands and classed as a nuclear receptor superfamily member. In humans, rabbits, rats and mice, it is found in large concentrations in the animals' colon, small intestine and liver. Quinalphos has been shown to interact with the NR1I2 protein, leading to increased activity [58].

Quinalphos demonstrates high‐affinity binding to several neurologically, hormonally and metabolically critical human proteins, suggesting a mechanistic basis for its toxicity. As mentioned in Table 2, it binds strongly to acetylcholinesterase (ACHE, ΔG = −8.2 kcal/mol), a key enzyme in neurotransmission, potentially causing neurotoxicity through cholinergic overstimulation. Similarly, its interactions with the androgen receptor (AR, ΔG = −7.1), oestrogen receptor alpha (ESR1, ΔG = −5.8) and 3β‐hydroxysteroid dehydrogenase type 3 (HSD3B3, ΔG = −7.68) indicate endocrine–disruptive potential by interfering with steroid hormone signalling and biosynthesis. In addition, quinalphos shows moderate binding to oxidative stress‐regulating enzymes such as catalase (CAT), glutathione reductase (GSR) and glucose‐6‐phosphate dehydrogenase (G6PD), implicating it in the disruption of redox homoeostasis and induction of oxidative stress. Furthermore, its affinity toward cytochrome P450 enzymes (e.g., CYP1A1 and CYP19A1) raises concerns about impaired xenobiotic metabolism and detoxification.

**TABLE 2:** Estimated binding affinity for free binding energy for various target proteins with quinalphos.

Gene	PDB ID	Average ΔG (kcal/mol)	Gene	PDB ID	Average ΔG (kcal/mol)
ACHE	4EY7	−8.2	CYP1A1	4I8V	−4.75
AHR	5V0L	−5.1	CYP19A1	3S79	−4.56
ALB	1AO6	−6.2	CYP26B1	1FAH	−4.92
AR	2Q7I	−7.1	G6PD	6E08	−5.8
ESR2	1YYE	−6.16	GSR	2GRT	−5.6
ESR1	3OS8	−5.8	CAT	1DGF	−5.6
BCHE	6SAM	−3.3	STAR	3P0L	−4.7
HSD3B3	1HXH	−7.68	NR1I2	1ILH	−6.02

Quinalphos (QP) demonstrates multisystem molecular toxicity in humans through high‐affinity binding to a wide range of proteins, disrupting neurological, endocrine, metabolic and detoxification pathways. QP targets acetylcholinesterase (ACHE), leading to the disruption of synaptic neurotransmission. This inhibition can result in neurobehavioral deficits and may contribute to neurodegeneration (link). It also exhibits oxidative stress, leading to testicular damage, including shrinkage of seminiferous tubules and degeneration of the germinal epithelium, which impairs spermatogenesis and testosterone synthesis. The depletion of protein content in organs such as the liver under quinalphos stress is attributed to impaired protein synthesis machinery and increased proteolysis, reflecting altered gene expression in protein metabolism and stress response. These effects are mediated through both direct protein targeting and downstream gene regulatory disturbances, significantly increasing the risk of disease and multisystem dysfunction in exposed individuals.

### 3.2 Gene Functional Regulatory Network

**FIGURE 1:**
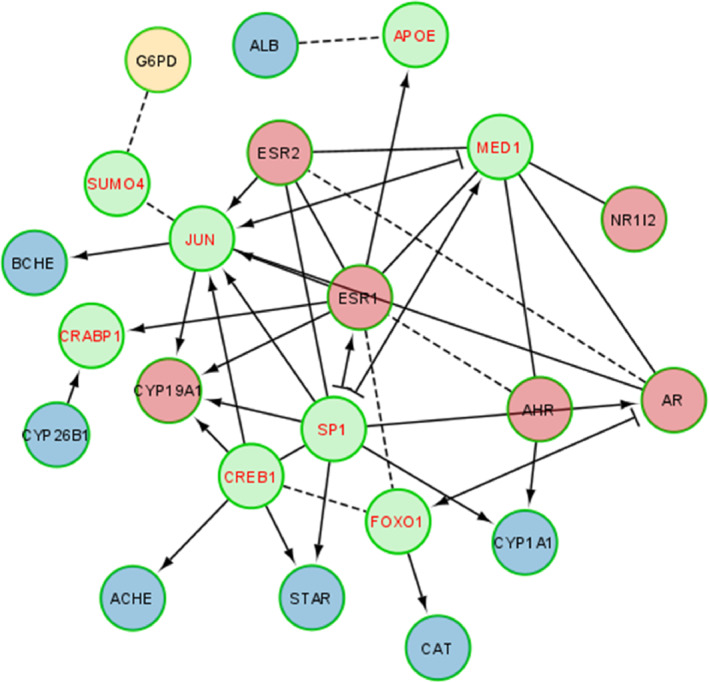
Gene regulatory network (red coloured nodes: increasing the affect, blue coloured nodes: decreasing the affect, green coloured nodes: linker genes).

The database CTD has enlisted 16 affected genes, where there are 9 top‐interacting genes (CAT, GSR, STAR, ACHE, BCHE, CYP26B1, G6PD, HSD3B3, NR1I2 and AHR) with quinalphos. Quinalphos exposure results in differential expression of genes (DEGs) (from the identified 16 nos.) as in a decrease in activity (down‐regulation) in 9 nos. (ACHE, ALB, BCHE, CAT, CYP1A1, CYP26B1, GSR, HSD3B3 and STAR) and increases in activity (up‐regulation) in 6 Nos. (AHR, AR, CYP19A1, ESR1, ESR2 and NR1I2). These genes are interrelated in their designated functions, as seen in the Figure 1 derived using Cytoscape software. This indicates the interaction of these differentially expressed genes associated with various regulatory factors, pathways and functions in the human body. Knowing their relationship allows the discovery of therapeutic targets (receptors, antagonists and enzyme modulators) that can reduce QP‐induced toxicity and block its interaction with significant pathways [59]. The gene regulatory network is created using the Reactome database to analyse the interaction between the affected expression of genes and exposure to quinalphos. Studies have identified significant associations between these genes and other genes, such as ESR1, AR and CREB1. ESR1 belongs to the nuclear receptor target family within the steroidal subfamily. Several pesticides exhibit oestrogenic and/or anti‐androgenic properties via the oestrogen receptor (ER) and/or androgen receptor (AR), and countless additional synthetic compounds may also contain similar oestrogenic and anti‐androgenic effects. The aryl hydrocarbon receptor (AHR) is a regulatory protein that governs the transcription of genes involved in the processing of exogenous compounds and the proliferation and differentiation of cells. The activity of the molecule is contingent upon the presence of binding molecules.

### 3.3 Molecular Docking Analysis

**FIGURE 2:**
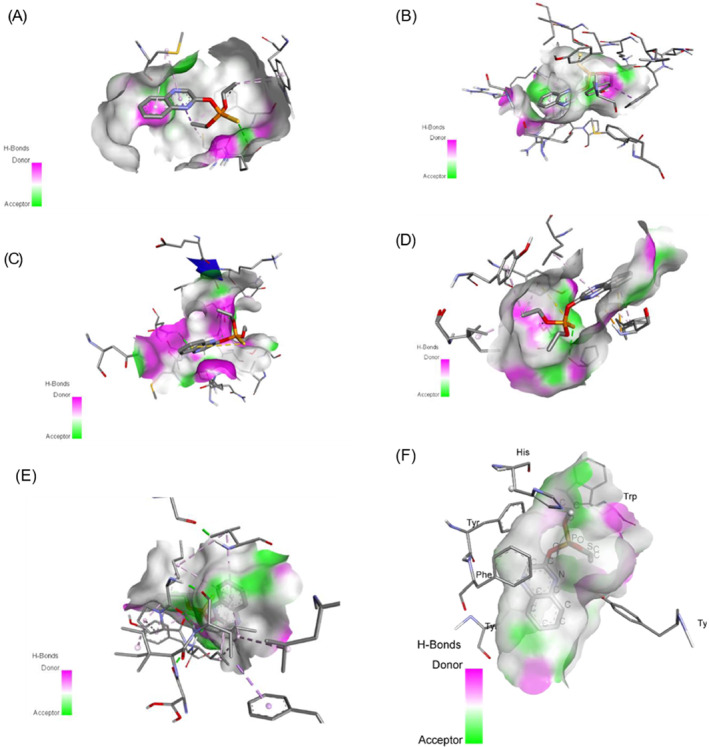
Molecular docking with quinalphos pesticide (A) NR1I2: **1ILH** (B) ESR2: **1YYE** (C) ALB: **1AO6** (D) AR: **2Q7I** (E) HSD3B3: **1HXH** (F) ACHE: **4EY7**.

The AutoDock‐based blind docking (BD) technique determines the appropriate protein‐coding genes with the maximum binding affinity with the quinalphos drug. The scoring function calculates a score for each position to rank the different poses and ligands. The docking process can be divided into two steps, namely, pose creation and scoring. Pose creation refers to the techniques used to generate different conformations of proteins and ligands and align different ligand conformations within the protein‐binding region. The scoring component is necessary to quantitatively assess posture quality during the docking process. Because docking is often used to screen large chemical libraries, quick pose creation and quality evaluation techniques that require minimal computational cost are necessary. Several streamlining measures are needed for the entire docking procedure to achieve this. To investigate the potential binding of pesticides, a thorough docking analysis of the genes is carried out and listed in Table 2, focusing on the expression of the differential proteins. The prepared compounds are subjected to docking with the target protein using AutoDock 4.2, generating hundreds of 10 conformations for each molecule. The “analysis” command in the docking parameter file prompts AutoDock to do a cluster analysis of the various docked conformations, explicitly focusing on the minimal energy obtained in each iteration. The coordinates are expressed in a modified PDB format, where four decimal values are added after the x‐, y‐, and z‐coordinates. These values represent the vdW + h bond + desolvation energy of the atom's interaction, the electrostatic interaction of the atom, the partial charge and the RMSD from the reference conformation. After completing 10 genetic algorithm runs, we calculated the mean value of the estimated free energy of binding (ΔG). The genes listed in ascending order of affinity are NR1I2, ESR2, ALB, AR, HSD3B3 and ACHE. Figure 2A–F depict the molecular docking of the quinalphos pesticide. The six remaining genes exhibited reduced affinity towards quinalphos, with CYP19A1 and BCHE displaying the lowest affinities, resembling the behaviour of the negative control. These targets are implicated in hormone signalling, detoxification and cellular stress response. Knowledge of these interactions makes it possible to rationally design therapeutic agents (receptor antagonists and enzyme modulators) that can neutralise QP‐induced toxicity or inhibit its interaction with essential pathways.

### 3.4 Genes‐TFs Regulatory Network

**FIGURE 3:**
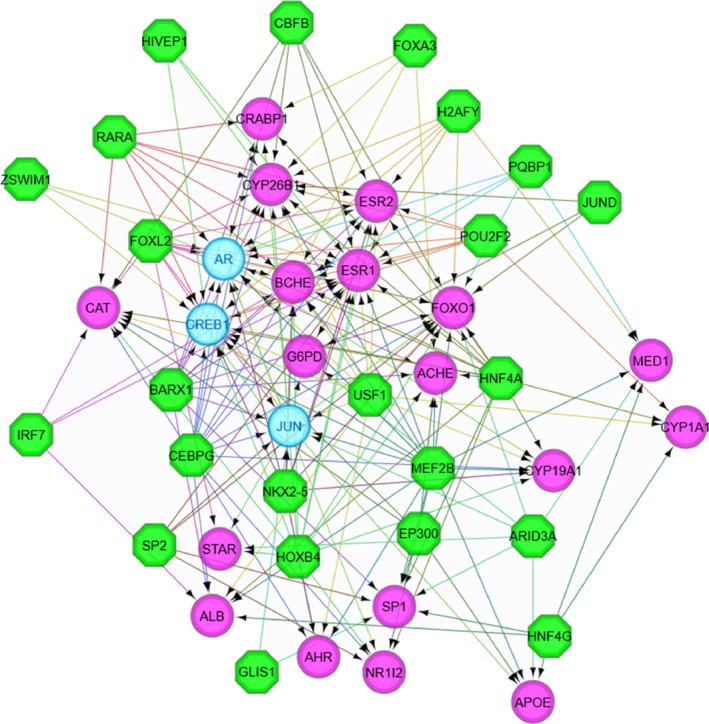
Gene TFs regulatory network (green nodes: regulators, purple nodes: regulated (targets), blue nodes: both regulators and targets).

To identify interactions of differentially upregulated and downregulated gene expression, a gene‐TFs regulatory network is created. Analysis of high‐degree nodes (those with a score of 10 or greater) identified 10 transcription factors (TFs), including MEF2B, HNF4A, CEBPG, USF1, BARX1, HOXB4 and FOXL2, as shown in Figure 3. The CYP26B1, ESR1 and BCHE, is regulated by the BARX1, CEBPG, FOXL2, HNF4A, HOXB4, MEF2B and USF1 transcription factors. QP decreases cytochrome P450 expression, and the down‐regulation of Cytochrome P450 in QP‐exposed animals is related to elevated LPO and H_2_O_2_ levels because free radicals react with lipids and increase lipid peroxidation. Cytochrome P450 enzymes in Leydig cells normally generate ROS during steroid hydroxylation. Environmental toxicants or metabolites act as pseudo‐substrates for CYP enzymes, resulting in enhanced ROS production via the CYP pseudo‐substrate‐O2–O_2_ complex. Most steroid enzymes are CYPs or HSDs. Cytochrome P450 catalyses the initial step in steroidogenesis, cholesterol side‐chain breakage. The first rate‐limiting and hormonally regulated step in steroid hormone synthesis is the mitochondrial conversion of cholesterol to pregnenolone [19]. Pesticides interact with oestrogen and androgen receptors to exert oestrogenic and anti‐androgenic effects. Quinalphos is an ER agonist. Agonists activate a receptor by binding to one inside or on the cell's surface [17]. It inhibits butyrylcholinesterase, albeit some require cytochrome P450 metabolism to an oxon form. Inhibition of AChE causes overstimulation at cholinergic synapses in the autonomic nervous system, neuromuscular junction and CNS [18].

### 3.5 Gene TF‐mRNA Regulatory Network

**FIGURE 4:**
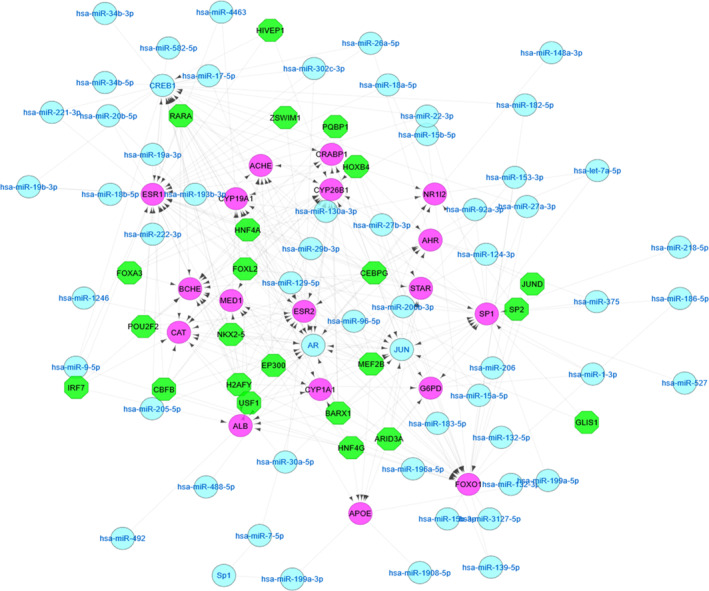
Gene TF‐mRNA regulatory network (green nodes: genes, blue nodes: miRNA, purple nodes: targets).

The TF‐ mRNA regulatory network is a complex network that regulates gene expression. Transcription factors bind to the promoter region of a gene and activate or repress its transcription. miRNAs regulate gene expression by binding to complementary sequences in mRNA transcripts, inhibiting translation, or promoting the degradation of mRNA transcripts. As stated in the methodology, the authors used CyTargetLinker tools and the ‘miRTarBase’ and ‘TargetScan’ databases to retrieve the experimentally validated 1566 miRNA targets that are responsible for the regulation of differentially expressed gene expression of quinalphos. Similarly, 683 miRNAs (very high) from the mirDP database and 393 from the mortar base database have been identified that are experimentally validated by any two methods, such as reporter assay, western blot, qPCR (strong evidence), microarray and NGS. pSLIAC, CLIP‐Seq (less strong evidence). Present study considers only 41 miRNA experimentally validated miRNAs by reporter assay, western blot and qPCR (strong evidence), a total of 14 genes have been found to be the 41 miRNA target, including STAR (hsa‐miR‐153‐3p, hsa‐miR‐1‐3p), NR1I2 (hsa‐miR‐18a‐5p, hsa‐miR‐148a‐3p, hsa‐let‐7a‐5p), G6PD (hsa‐miR‐206, hsa‐miR‐1‐3p), ESR2 (hsa‐miR‐92a‐3p, hsa‐miR‐30a‐5p, hsa‐miR‐302c‐3p, hsa‐miR‐20b‐5p, hsa‐miR‐17‐5p), ESR1 (“hsa‐miR‐9‐5p, hsa‐miR‐4463, hsa‐miR‐302c‐3p, hsa‐miR‐29b‐3p, hsa‐miR‐26a‐5p, hsa‐miR‐222‐3p, hsa‐miR‐221‐3p, hsa‐miR‐22‐3p, hsa‐miR‐20b‐5p, hsa‐miR‐206, hsa‐miR‐19b‐3p, hsa‐miR‐19a‐3p, hsa‐miR‐193b‐3p, hsa‐miR‐18b‐5p, hsa‐miR‐18a‐5p, hsa‐miR‐130a‐3p, hsa‐miR‐129‐5p”), CYP26B1 (hsa‐miR‐15b‐5p, hsa‐miR‐15a‐5p), CYP1A1 (hsa‐miR‐200b‐3p), CYP19A1 (hsa‐miR‐4463, hsa‐miR‐19b‐3p), CAT (hsa‐miR‐30a‐5p), BCHE (hsa‐miR‐26b‐5p, hsa‐miR‐335‐5p), AR (hsa‐miR‐7‐5p, hsa‐miR‐488‐5p, hsa‐miR‐205‐5p, hsa‐miR‐1246, hsa‐miR‐124‐3p), ALB (hsa‐miR‐492), AHR (hsa‐miR‐375, hsa‐miR‐26a‐5p, hsa‐miR‐124‐3p, hsa‐let‐7a‐5p) and ACHE (hsa‐miR‐212‐3p) as shown in Figure 4. Table 3 displays details on transcription regulators, miRNA and regulated genes.

**TABLE 3:** Transcription factor and gene regulation.

Gene	miRNA	TFs	Expression
CYP1A1	hsa‐miR‐200b‐3p	POU2F2, HNF4G, HNF4A, USF1	Down
ESR1	hsa‐miR‐9‐5p, hsa‐miR‐4463, hsa‐miR‐302c‐3p, hsa‐miR‐29b‐3p, hsa‐miR‐26a‐5p, hsa‐miR‐222‐3p, hsa‐miR‐221‐3p, hsa‐miR‐22‐3p, hsa‐miR‐20b‐5p, hsa‐miR‐206, hsa‐miR‐19b‐3p, hsa‐miR‐19a‐3p, hsa‐miR‐193b‐3p, hsa‐miR‐18b‐5p, hsa‐miR‐18a‐5p, hsa‐miR‐130a‐3p, hsa‐miR‐129‐5p	PQBP1, CBFB, H2AFY, NKX2‐5, SP2, POU2F2, CEBPG, BARX1, HIVEP1, ZSWIM1, MEF2B, USF1, HNF4A, RARA, FOXL2, IRF7, HOXB4, CREB1	Up
CYP19A1	hsa‐miR‐200b‐3p	NKX2‐5, CEBPG, MEF2B, HNF4A, USF1, HOXB4, AR	Up
ESR2	hsa‐miR‐92a‐3p, hsa‐miR‐30a‐5p, hsa‐miR‐302c‐3p, hsa‐miR‐20b‐5p, hsa‐miR‐17‐5p	CBFB, H2AFY, NKX2‐5, POU2F2, CEBPG, BARX1, FOXL2, USF1, HNF4A, RARA, HOXB4, AR	Up
G6PD	hsa‐miR‐206, hsa‐miR‐1‐3p	H2AFY, JUND, ARID3A, JUN	Affects the activity
AHR	hsa‐miR‐375, hsa‐miR‐26a‐5p, hsa‐miR‐124‐3p, hsa‐let‐7a‐5p	NKX2‐5, CEBPG, MEF2B, USF1, HOXB4	Up
AR	hsa‐miR‐7‐5p, hsa‐miR‐488‐5p, hsa‐miR‐205‐5p, hsa‐miR‐1246, hsa‐miR‐124‐3p	PQBP1, CBFB, H2AFY, NKX2‐5, POU2F2, CEBPG, BARX1, MEF2B, ZSWIM1, USF1, HNF4A, RARA, FOXL2, AR, JUN	Up
NR1I2	hsa‐miR‐18a‐5p, hsa‐miR‐148a‐3p, hsa‐let‐7a‐5p	SP2, CEBPG, MEF2B, HNF4A, HOXB4, AR	Up
ALB	hsa‐miR‐492	CEBPG, BARX1, HNF4G, MEF2B, HNF4A, IRF7, USF1	Down
STAR	hsa‐miR‐153‐3p, hsa‐miR‐1‐3p	CEBPG, BARX1, HNF4G, MEF2B, HNF4A, IRF7, USF1	Up
CAT	hsa‐miR‐30a‐5p	HOXB4, MEF2B, RARA, FOXL2, CEBPG, HNF4A, EP300, USF1, IRF7, CBFB	Down
BCHE	hsa‐miR‐26b‐5p, hsa‐miR‐335‐5p	NKX2‐5, HOXB4, MEF2B, RARA, FOXL2, CEBPG, POU2F2, HNF4A, FOXA3, USF1, JUN, PQBP1, BARX1, IRF7	Down
ACHE	hsa‐miR‐212‐3p	HOXB4, MEF2B, FOXL2, ACHE, HNF4A, FOXA3, EP300, BARX1	Down

### 3.6 Network Enrichment Analysis

#### 3.6.1 Gene Disease Enrichment

**FIGURE 5:**
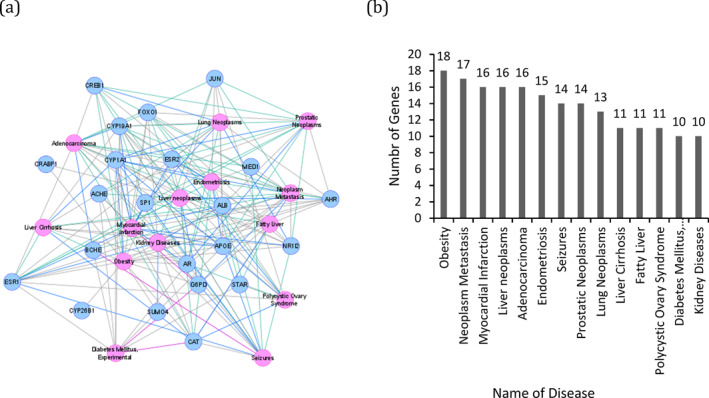
Disease enrichment network (a) number of genes with disease association (purple nodes: disease, blue nodes: gene IDs) (b) bar graph of the number of genes associated with various diseases.

One of the most critical aspects of this research is the identification of genes associated with a given disease. This process is called enrichment analysis and can be performed by examining a list of genes (Table 1) associated with the disease in question. The goal of gene enrichment analysis is to identify a subset of genes that are overrepresented in each set. For example, when a gene associated with breast cancer is investigated, one needs to identify those that appear more frequently than expected from the reference gene list and then look for an explanation for why this might occur. In the present analysis with the quinalphos exposure database, gene–disease enrichment analysis revealed that the DEGs are responsible for 14 human diseases, as shown in Figure 5a. This figure shows a gene–disease interaction network, where blue nodes represent genes and pink nodes represent diseases. The connecting lines indicate known associations between genes and various diseases based on the literature evidence. Similarly, Figure 5b shows the number of genes associated with multiple diseases. The highest association with the disease included **obesity, neoplasm metastasis, myocardial infarction, liver neoplasms, adenocarcinoma and endometriosis.** The aryl hydrocarbon receptor (AHR) is a ligand‐activated transcription factor highly expressed in hepatocytes. AHR deficiency significantly reduces weight gain and adiposity and increases multilocular lipid droplet formation within perigonadal white adipose tissue [60]. Similarly, a study investigated the role of PCB‐77, a ligand of the aryl hydrocarbon receptor (AhR), in reducing obesity. It found that AhR‐deficient female mice were resistant to diet‐induced obesity. During weight loss, PCB‐77 negatively affected glucose tolerance in male mice. However, in obese males losing weight, the absence of the AhR gene reversed the negative impact of PCB‐77 on glucose tolerance, whereas in females, it worsened glucose intolerance. Additionally, in female mice, IRS2 mRNA levels in adipose tissue were lower than those in vehicle (VEH)‐treated controls [61]. Another genome‐wide association study (GWAS) identified > 250 loci for body mass index (BMI). Most GWAS loci represent clusters of common noncoding variants. In the present analysis, data from 718,734 individuals are combined to identify rare and low‐frequency coding variants associated with BMI. From the 14 coding variants in 13 genes, of which 8 variants are in genes (ZBTB7B, ACHE, RAPGEF3, RAB21, ZFHX3, ENTPD6, ZFR2 and ZNF169) newly implicated in human obesity, 2 variants are in genes (MC4R and KSR2) previously observed to be mutated in extreme obesity and 2 variants are in GIPR [62].

#### 3.6.2 Gene Ontology Enrichment

**FIGURE 6:**
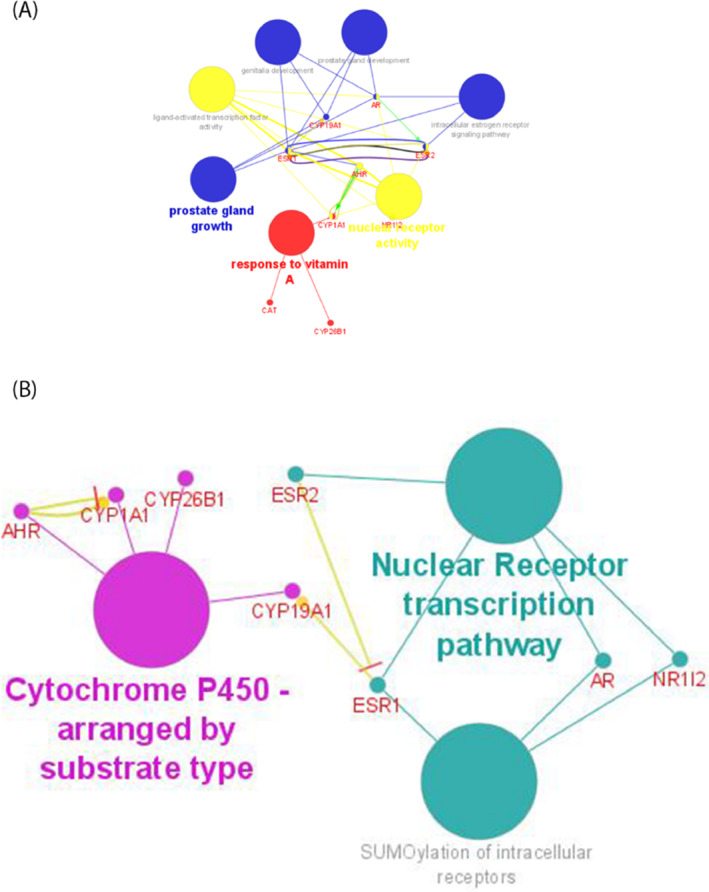
Gene ontology analysis. (A) Functionally grouped network with terms as nodes linked based on their kappa score level (≥ 0.3), where only the label of the most significant term per group is shown. The node size represents the term enrichment significance. (B) Biological pathway.

To obtain an overview of the molecular functions and processes of the 16 DEGs in the gene‐disease association network, we performed functional and pathway enrichment analyses using ClueGO. Gene ontology (GO) considers three distinct aspects of gene function. They are divided into three main categories: biological processes (BP), molecular functions (MF) and cellular components (CC). Network‐based pathway enrichment analysis is used to identify regulatory pathways or gene ontologies with statistically significant associations with a given gene set. The idea behind enrichment analyses is that if a gene set linked to a pathological condition is enriched in a given pathway or gene ontology, it may suggest a connection between the pathway and the disease. Among the GO BP terms having the highest significance, we find some linked to genitalia development, intracellular oestrogenic receptor signalling pathway, ligand‐activated transcription factor activity, nuclear receptor activity, prostate gland development, prostate gland growth and response to vitamin A, as shown in Figure 6A. Quinalphos‐induced genes ESR1, ESR2, AR (Upregulated), STAR and CYP1A1 (downregulated) are associated with oestrogen signal pathway and metabolic process, respectively. The activation of oestrogen receptors (ERs) and signal transduction is the principal focus of the oestrogen signal pathway. The binding of oestrogen to ERs or ER elements along with specific NRs influences the activity of the target gene regulatory region, enhancing regulation of gene expression and Oestrogen signalling pathway, and also various diseases, including breast cancer, gastric cancer and atherosclerosis, are related [63, 64, 65, 66], vitamin A is allied with many molecular mechanisms such as the regulation core of gene expression, activation of kinase cascades, RA signalling associated with NRs, nucleic acids response elements, retinoic acid (RA) response elements, multiple coregulators etc, and therefore essential throughout the initiation of life [67, 68, 69]. BCHE, CYP1A1 (downregulated) and NR1I2 (upregulated) genes are involved in the drug metabolic process. Issues of pregnancy complications, oxidative stress, proliferation inhibition, DNA damage and many more are related to drug metabolisms to differential expression of these genes [65, 70, 71, 72, 73].

The present study adopted a novel approach to examine the association of genes with well‐described signalling for metabolic and regulatory pathways. ClueGO retrieved associations between genes from curated databases such as KEGG [74] and REACTOME [75]. The analysis detected 3 pathways that showed significant disease enrichment, as shown in Figure 6B. Interestingly, the pathways belonged to several categories linked to Cytochrome P450—arranged by substrate type, nuclear receptor transcription pathway and SUMOylation of intracellular receptors. Nuclear receptors (NRs) are prevalent in preventing several diseases caused by foreign elements through their detoxification sense and response. NRs are, in general, DNA‐binding bifunctional transcription factors (TFs) that link to specific cellular molecules such as hormones, vitamins etc. It is observed that quinalphos affected genes, namely ESR2, ESR1, AR and NR1I2, are actively associated with nuclear receptor transcription activities and pathways. In contrast, the latter three genes are associated with SUMOylation (activity of small ubiquitin‐like modifier proteins) of the intracellular receptor [76] quantitative investigations have demonstrated that quinalphos exposure in humans is associated with a statistically significant suppression of total T3 levels (*p* < 0.01), a marginal decrease in T4 (about 7%), and elevated TSH levels (about 28% higher than controls), indicating impaired thyroid function [77]. In animal models, quinalphos induces oxidative stress and liver injury, which can disrupt the metabolism of vitamins A and D [67, 70, 71, 78]. SUMOylation is responsible for several activities associated with NRs, such as transcriptional repression and altered NR signalling [3, 65, 76, 79]. The association is shown in Figure 6B, where the most significant pathways are depicted with the largest node size.

### 3.7 Gene‐Drug Interaction Network

**FIGURE 7:**
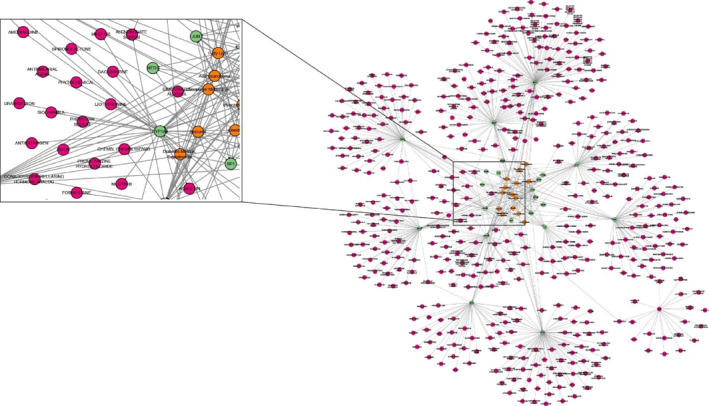
Gene–drug–disease interaction network regulated by several drugs. In this network (orange nodes: disease, red nodes: drug, green node: gene).

The present study includes investigations of 16 different protein‐coding genes to predict interactions between drugs and proteins. The analysis identified 1445 drugs for 15 genes; the network was pruned by applying the threshold value 0.1, and finally, 446 drug therapeutic targets were obtained, as shown in Figure 7. Gene HSD3B3 has not been predicted to regulate any drug expression. The drug has been categorised into two groups to assess the translation relevance of gene–drug interaction: approved and not approved (experimental and investigational) compounds. The ACHE gene exhibited a higher number of drug interactions (*n* = 64), with 31 approved and 33 non‐approved drugs for 7 diseases. Similarly, the ESRI found extensive interaction (*n* = 90), but a larger fraction (*n* = 55) was unapproved for 13 diseases, indicating a research‐heavy focus. In contrast, genes such as G6PD and GSR demonstrated a higher proportion of approved drugs (71% and 79%, respectively), suggesting more established therapeutic applications. A summary of the approved and non‐approved drugs is presented in Table 4.

**TABLE 4:** List of genes and their predicted drug for the disease.

Gene	Predicted drug		Predicted disease
	Approved	Not approved	
ACHE	Ambenonium, pralidoxime chloride, demecarium bromide, magnesium chloride anhydrous, edrophonium cation, pralidoxime cation, neostigmine, isoflurophate, ambenonium chloride, echothiophate iodide, edrophonium chloride, donepezil hydrochloride, physostigmine salicylate, hexafluorenium bromide, echothiophate, distigmine, galantamine, tacrine, donepezil, rivastigmine, human calcitonin, salmon calcitonin, pyridostigmine bromide, rivastigmine tartrate, tacrine hydrochloride, propanidid, physostigmine, pyridostigmine, betamethasone, malathion, pramlintide	Huperzine A, acotiamide hydrochloride, galactitol, turbinatine, desoxycordifoline, radiosensitizing agent, mimopezil, adrenomedullin, &beta;‐CGRP, amylin, adrenomedullin 2/intermedin, AC187, methanesulfonyl fluoride, KBP‐088, [125I]CT (human), buntanetap, acotiamide, amocarzine, CT‐(8–32) (salmon), KBP‐066, &alpha;‐CGRP, NN1213, [125I]CT (salmon), itopride, eptastigmine, suronacrine maleate, amlintide, ladostigil, phenserine, 6‐aminonicotinamide, 8‐azaguanine, davalintide, skimmianine	Lung neoplasms, obesity, myocardial infarction, diabetes mellitus, liver cirrhosis, seizures, prostatic neoplasms
AHR	Methylcellulose, tapinarof, romiplostim, flavone, thiabendazole, carbaril	Quinalphos, CHEMBL:CHEMBL541230, benzo[def]chrysene, beta‐naphthoflavone, CHEMBL:CHEMBL597452, CHEMBL:CHEMBL57394, linuron, desmedipham, CHEMBL:CHEMBL295337, chrysene, salicylanilide, pyraflufen‐ethyl, rimsulfuron, methabenzthiazuron, diflubenzuron, CSC, cyprodinil, CHEMBL:CHEMBL1873684, aldicarb, dichlon, diphenylurea, yangonin, benzofuran, dimethyl yellow, CHEMBL:CHEMBL70582, desmethoxyyangonin, CHEMBL:CHEMBL77675, 1‐naphthylisothiocyanate, CHEMBL:CHEMBL538965, 3‐methylcholanthrene, naphthoquinone, dibenz[a,h]anthracene, questiomycin B, CHEMBL:CHEMBL84685, benzo[b]fluoranthene, 2‐chloro‐5‐nitro‐n‐phenylbenzamide, colforsin	Lung neoplasms, obesity, endometriosis, myocardial infarction, kidney diseases, fatty liver, adenocarcinoma, liver cirrhosis, prostatic neoplasms, neoplasm metastasis
ALB	GADOFOSVESET trisodium, GADOBENATE dimeglumine, bismuth, AMODIAQUINE hydrochloride, ALBUMIN human‐KJDA, OLMESARTAN medoxomil, SODIUM DODECYL sulfate, raltitrexed, fluconazole, phenylbutazone, pyrogallol, NAPROXEN SODIUM	Cochinchinenin C, CHEMBL:CHEMBL1945287, urocortin 3, [125I]antisauvagine, antisauvagine, epigallocatechin, iodipamide, K31440, K41498, [19F]‐haloperidol, urocortin 2, sauvagine, urocortin 1, [125I]astressin, [125I]urocortin 1 (mouse, rat), astressin 2B, astressin, urotensin 1 (fish), [125I]sauvagine (frog), urocortin 1, EPICATECHIN gallate, corticotrophin‐releasing hormone, corticotropin‐releasing factor, Evans blue, epicatechin, epigallocatechin‐3‐gallate	Lung and lever neoplasms, obesity, endometriosis, myocardial infarction, fatty liver, kidney diseases, polycystic ovary syndrome, adenocarcinoma, liver cirrhosis, seizures, prostatic neoplasms
AR	Apalutamide, oxandrolone, dromostanolone, methandrostenolone	Enobosarm, GLPG0492, APC‐100, CHEMBL:CHEMBL1099140, CHEMBL:CHEMBL1738937, CHEMBL:CHEMBL1946449, LGD‐2941, CHEMBL:CHEMBL1923999, CHEMBL:CHEMBL1946014, (R)‐BICALUTAMIDE, CHEMBL:CHEMBL1738936, hydroxyflutamide, CHEMBL:CHEMBL1946013, MK‐0773	Seizures, endometriosis, obesity, liver cirrhosis, lung neoplasms, polycystic ovary, syndrome, prostatic neoplasms, liver neoplasms, adenocarcinoma, neoplasm metastasis, myocardial infarction, diabetes mellitus
BCHE	Succinylcholine, mivacurium, demecarium bromide, echothiophate iodide, isoflurophate, meptazinol, tacrine hydrochloride, volanesorsen, propanidid, rivastigmine tartrate, rivastigmine, donepezil, galantamine, trifarotene, memantine hydrochloride, tamibarotene, bupivacaine, tazarotene, triamcinolone, adapalene	Hexafluorenium, BMS753, BMS‐189532, RO 41‐5253, AGN193836, BMS614, eptastigmine, AGN193109, CD666, immunotoxin, phenserine, ANESTHETIC agent, RO 40‐6055, BMS493, phenothiazine	Obesity, myocardial infarction, diabetes mellitus, adenocarcinoma, liver cirrhosis, seizures, neoplasm, metastasis
CAT	Dehydrated alcohol	None	Liver neoplasms, obesity, endometriosis, myocardial infarction, kidney diseases, fatty liver, diabetes mellitus, adenocarcinoma, polycystic ovary, syndrome, liver cirrhosis, seizures, neoplasm metastasis
CYP19A1	Testolactone, testosterone enanthate, aminoglutethimide, letrozole, isopropyl alcohol, anastrozole, aztreonam, exemestane, alendronate sodium, captopril, hydroquinone, atropine	CHEMBL:CHEMBL572637, CHEMBL:CHEMBL1077603, MPI‐674, gonadotropin‐releasing hormone analog, fadrozole hydrochloride, atamestane, environmental oestrogen, antifungal agent, liarozole, anabolic steroid, phytoestrogen, bioflavonoid, formestane, opiate, androstenedione, adenovirus vector, naringenin, RECOMBINANT tumour necrosis factor family protein, fadrozole, nonsteroidal antiinflammatory drug	Lung and liver neoplasms, obesity, endometriosis, myocardial infarction, fatty liver, kidney diseases, diabetes mellitus, polycystic ovary syndrome, adenocarcinoma, liver cirrhosis, seizures, prostatic neoplasms, neoplasm metastasis
CYP1A1	Rutin, granisetron, dacarbazine, nilotinib, spironolactone, PROMETHAZINE hydrochloride, phenobarbital, PHENYTOIN sodium, lansoprazole, carbamazepine, LIOTHYRONINE	Antimalarial agent, biochanin A, phytochemical, MT47‐100, amodiaquine, hypericin, antiestrogen, acacetin, anticonvulsant agent	Lung and liver neoplasms, obesity, endometriosis, myocardial infarction, diabetes mellitus, adenocarcinoma, polycystic ovary syndrome, liver cirrhosis, seizures, prostatic neoplasms, neoplasm metastasis
CYP26B1	None	Talarozole	Obesity
ESR1	Elacestrant, oestriol, oestrogens, esterified (USP), BORIC acid, ethylenediamine, oestradiol cypionate, lasofoxifene, megestrol, elacestrant hydrochloride, acrylamide, iodine, oestrogen, estetrol, fulvestrant, quinestrol, chlorotrianisene, clomiphene, dienestrol, estropipate, ospemifene, Oestrogens, conjugated (USP), synthetic conjugated oestrogens, B, lasofoxifene tartrate, ethynodiol diacetate, raloxifen, desogestrel, cyclofenil, palmitate, dienogest, clomiphene citrate, benzododecinium chloride, oestrogens, conjugated synthetic A, toremifene, oestrone, mestranol	Brilanestrant, CHEMBL:CHEMBL1091137, selective oestrogen receptor degrader AZD9496, 4‐triethylgermylphenol, CHEMBL:CHEMBL2313444, CHEMBL:CHEMBL2071180, glycitein, 1‐hydroxy‐6‐(4‐hydroxy‐phenyl)‐1‐phenyl‐hexan‐3‐one (enantiomeric mix), CHEMBL:CHEMBL2313445, arzoxifene, endoxifen, selective oestrogen receptor modulator CC‐8490, GTX‐758, icaritin, promestriene, CHEMBL:CHEMBL384441, methylisothiocyanate, fispemifene, ICI‐164384, hormone therapy agent, MK‐6913, monoethanolamine, giredestrant, alfadex, fosfestrol, n‐propyl bromide, acolbifene, selective oestrogen receptor covalent antagonist H3B‐6545, selective oestrogen receptor modulator TAS‐108, CHEMBL:CHEMBL1432305, CHEMBL:CHEMBL354663, amcenestrant, 4‐methylbenzoic acid methyl ester, sulfentrazone, SR16234, TTC‐352, puerarin, AMI‐1, gestrinone, triadimefon, camizestrant, methiocarb, sivifene, arsanilic acid, CHEMBL:CHEMBL503990, CHF4227, PCC0208017, diethylstilbestrol dipropionate, CHF 4227, LY2245461, compound 14 [PMID: 34333981], TAS‐108, nomegestrol acetate, vintafolide, alfatradiol	Lung and liber neoplasms, obesity, endometriosis, myocardial infarction, fatty liver, kidney diseases, diabetes mellitus, polycystic ovary syndrome, Adenocarcinoma. Liver cirrhosis, seizures, prostatic neoplasms
ESR2	OEstrogen, lasofoxifene, ospemifene, synthetic conjugated oestrogens, B, estetrol, raloxifen, lasofoxifene tartrate, estramustine phosphate sodium, oestrogens, esterified (USP), oestrogens, conjugated synthetic a, oestriol, bazedoxifene, bazedoxifene acetate, allylestrenol, diethylstilbestrol diphosphate, fam‐trastuzumab deruxtecan‐nxki, cyclofenil, toremifene, oestrogens, conjugated (USP), fulvestrant, quinestrol, chlorotrianisene, Alendronate sodium, trilostane	AUS‐131, MF101, erteberel, CHEMBL:CHEMBL2332580, prinaberel, MK‐6913, arzoxifene, PHTPP, FERB 033, ICI‐164384, fispemifene, menerba, diarylpropionitrile, CHF4227, WAY200070, acolbifene, (s)‐liquiritigenin, PCC0208017, R,R‐THC, sivifene, LY2245461, compound 14 [PMID: 34333981], CHF 4227, TAS‐108, CR 1447, BISPHENOL A, AC‐186, HPTE, norendoxifen, idoxifene, PCC0105003, 4‐hydroxytamoxifen, droloxifene	Lung and liver neoplasms, obesity, endometriosis, myocardial infarction, adenocarcinoma, polycystic ovary, syndrome, prostatic neoplasms, neoplasm metastasis
G6PD	Rasburicase, phenazopyridine, pegloticase, sulfanilamide, sodium ascorbate, tafenoquine, sodium sulphate anhydrous, sulfadiazine, nalidixic acid, sulfacetamide, sodium nitrate, articaine, mafenide, sodium nitrite, nitrofurantoin, macrocrystals, sulfisoxazole, potassium chloride, ropivacaine, zinc chloride, dimercaprol, sulfamethoxazole, furazolidone, sulfamethazine, nitrofurazone, norethindrone acetate, tolazamide, succimer, glipizide, moxifloxacin, norfloxacin, glimepiride, tolbutamide, quinine, atazanavir, glyburide, gliclazide	Pamaquine, co‐trimoxazole, sitamaquine, DRUGSATFDA.NDA:021717, metabutethamine, carbasalate calcium, amodiaquine, acetaminophen/codeine, penicillin, epiandrosterone, triapine, articaine/epinephrine, acetaminophen/tramadol, PTHRP, nicorandil	Liver neoplasms, obesity, myocardial infarction, kidney diseases, fatty liver, diabetes mellitus, adenocarcinoma, seizures, neoplasm metastasis
GSR	Oxiglutatione, amifostine anhydrous, levofloxacin anhydrous, carmustine, goserelin acetate, mechlorethamine, ofloxacin, cefotaxime sodium, vitamin E, selenium, enalapril maleate, ondansetron hydrochloride, carvedilol, vitamin A, lansoprazole	NOV‐002, misonidazole, oral contraceptive, thyroxine	Not found
NR1I2	Rifaximin, menatetrenone, nafcillin, rifabutin, penicillin V, oxiconazole, demeclocycline, hypoxis hemerocallidea root extract, ethambutol hydrochloride, etonogestrel, rifapentine, dicloxacillin, dolutegravir sodium, cefadroxil anhydrous, terbinafine, sulfamethazine, griseofulvin, cephradine, sulfisoxazole, bosentan anhydrous, cefuroxime axetil, rifampin, isoniazid, memantine hydrochloride, floxacillin	solomonsterol A, solomonsterol B, theonellasterol H, theonellasterol D, conicasterol E, theonellasterol B, conicasterol B, theonellasterol F, conicasterol D, theonellasterol G, suvanine's sodium salt, theonellasterol E, theonellasterol C, ginsenoside RG3, CHEMBL: CHEMBL535714, navtemadlin	Lung and neoplasms, obesity, endometriosis, myocardial infarction, fatty liver, adenocarcinoma, liver cirrhosis, seizures, prostatic neoplasms, neoplasm metastasis
STAR	None	BDNF	Endometriosis, fatty liver, diabetes mellitus, polycystic ovary syndrome

## 4 Conclusion

Quinalphos is a widely applied organophosphate pesticide due to its high efficacy in pest control. However, it also carries potential health risks to humans, as exposure to quinalphos has been associated with alterations in the expression of 16 genes, which are linked to several health issues such as obesity, neoplasm metastasis, myocardial infarction, liver neoplasms, adenocarcinoma, endometriosis and so on. It has been demonstrated that 26 transcription factors mainly regulate the expression of these genes and could be targeted by 41 miRNAs. Additionally, the expression of genes is influenced by changes in the methylation status of DNA in the gene promoter region, known as CpG islands, which have a high methylation status and are less likely to be expressed than regions with a low methylation status. In light of this evidence, further research might help to understand the effects of quinalphos exposure on human health. These results suggest that quinalphos has the potential to cause significant health issues in humans and should be used with caution. Future research on these AI and deep learning approaches could significantly enhance the resolution and predictive power of QP toxicity studies. By leveraging large environmental and biomedical datasets, future research can move toward more precise risk assessment, early biomarker discovery and identification of novel therapeutic targets.

## Author Contributions

**Jyoti Kant Choudhari:** conceptualization, data curation, investigation, methodology, resources, software, validation, visualization, writing – original draft, writing – review and editing. **Biju Prava Sahariah:** conceptualization, data curation, investigation, methodology, writing – original draft, writing – review and editing. **Anand Kumar Jayapal:** conceptualization, methodology, writing – original draft, writing – review and editing. **Jyotsna Choubey:** conceptualization, investigation, methodology, writing – original draft, writing – review and editing. **Abhishek Tripathi:** methodology, writing – original draft, writing – review and editing.

## Funding

The authors have nothing to report.

## Ethics Statement

The authors have nothing to report.

## Consent

All authors have given their consent for publication.

## Conflicts of Interest

The authors declare no conflicts of interest.

## Permission to Reproduce Material From Other Sources

The authors have nothing to report.

## Data Availability

Data will be made available on request by corresponding authors.

## References

[1] S. Kumari , R. Dcunha , S. P. Sanghvi , et al., “Organophosphorus Pesticide Quinalphos (Ekalux 25 E.C.) Reduces Sperm Functional Competence and Decreases the Fertilisation Potential in Swiss Albino Mice,” Andrologia 53, no. 8 (2021): e14115, 10.1111/and.14115. 34014595

[2] V. P. Androutsopoulos , A. F. Hernandez , J. Liesivuori , and A. M. Tsatsakis , “A Mechanistic Overview of Health Associated Effects of Low Levels of Organochlorine and Organophosphorous Pesticides,” Toxicology 307 (2013): 89–94, 10.1016/j.tox.2012.09.011. 23041710

[3] D. Hemalatha , B. Nataraj , B. Rangasamy , K. Maharajan , and M. Ramesh , “Exploring the Sublethal Genotoxic Effects of Class II Organophosphorus Insecticide Quinalphos on Freshwater Fish Cyprinus carpio,” Journal of Oceanology and Limnology 39, no. 2 (2020): 661–670, 10.1007/s00343-019-9104-y.

[4] V. Subba Reddy Gangireddygari , D. Kanderi , R. Golla , et al., “Biodegradation of Quinalphos by a Soil Bacterium‐Bacillus Subtilis,” Pakistan Journal of Biological Sciences: PJBS 20, no. 8 (2017): 410–422, 10.3923/pjbs.2017.410.422. 29023062

[5] P. U. Sanganalmath , P. M. Nagaraju , and K. Sreeramulu , “Determination of Quinalphos in Human Whole Blood Samples by High‐Performance Thin‐Layer Chromatography for Forensic Applications,” Journal of Chromatography, A 1594 (2019): 181–189, 10.1016/j.chroma.2019.02.003. 30745138

[6] S. Ambreen and A. Yasmin , “Novel Degradation Pathways for Chlorpyrifos and 3, 5, 6‐Trichloro‐2‐Pyridinol Degradation by Bacterial Strain Bacillus thuringiensis MB497 Isolated From Agricultural Fields of Mianwali, Pakistan,” Pesticide Biochemistry and Physiology 172 (2020): 104750, 10.1016/j.pestbp.2020.104750. 33518043

[7] C. C. Lerro , S. Koutros , G. Andreotti , et al., “Organophosphate Insecticide Use and Cancer Incidence Among Spouses of Pesticide Applicators in the Agricultural Health Study,” Occupational and Environmental Medicine 72, no. 10 (2015): 736–744, 10.1136/oemed-2014-102798. 26150671 PMC4909328

[8] N. I. Kaur Dhanjal , P. Kaur , D. Sud , and S. S. Cameotra , “Persistence and Biodegradation of Quinalphos Using Soil Microbes,” Water Environment Research 86, no. 5 (2014): 457–461, 10.2175/106143013X13706200598514. 24961072

[9] C. Gonçalves , A. Dimou , V. Sakkas , M. F. Alpendurada , and T. A. Albanis , “Photolytic Degradation of Quinalphos in Natural Waters and on Soil Matrices Under Simulated Solar Irradiation,” Chemosphere 64, no. 8 (2006): 1375–1382, 10.1016/j.chemosphere.2005.12.020. 16469359

[10] I. M. Sadiqul , S. M. Kabir , Z. Ferdous , K. M. Mansura , and R. Md. Khalilur , “Chronic Exposure to Quinalphos Shows Biochemical Changes and Genotoxicty in Erythrocytes of Silver Barb, Barbonymus gonionotus,” Interdisciplinary Toxicology 10, no. 3 (2017): 99–106, 10.1515/intox-2017-0016. 30174533 PMC6107646

[11] J. K. Choudhari , B. P. Sahariah , J. K. Choubey , A. Patel , and M. K. Verma , “Identification of Potential Transcription Factor and Protein Kinases for Regulation of Differentially Expressed Genes for Fluoride Exposure in Human Using Expression2Kinases (X2K) Approach,” Netw Model Anal Health Inform Bioinforma 6, no. 1 (2017): 7, 10.1007/s13721-017-0148-7.

[12] J. K. Choudhari , J. Choubey , M. K. Verma , et al., Chromium Genotoxicity Associated With Respiratory Disease (IntechOpen, 2021).

[13] A. P. Davis , C. J. Grondin , R. J. Johnson , et al., “Comparative Toxicogenomics Database (CTD): Update 2021,” Nucleic Acids Research 49, no. D1 (2021): D1138–D1143, 10.1093/nar/gkaa891. 33068428 PMC7779006

[14] A. P. Davis , T. C. Wiegers , R. J. Johnson , D. Sciaky , J. Wiegers , and C. J. Mattingly , “Comparative Toxicogenomics Database (CTD): Update 2023,” Nucleic Acids Research 51, no. D1 (2022): D1257–D1262, 10.1093/nar/gkac833. PMC9825590 36169237

[15] P. Singh , P. K. Verma , R. Raina , S. Sood , and P. Sharma , “Maximum Contaminant Level of Arsenic in Drinking Water Potentiates Quinalphos‐Induced Renal Damage on Co‐Administration of Both Arsenic and Quinalphos in Wistar Rats,” Environmental Science & Pollution Research 27, no. 17 (2020): 21331–21340, 10.1007/s11356-020-08643-1. 32270456

[16] S. Takeuchi , M. Iida , H. Yabushita , T. Matsuda , and H. Kojima , “In Vitro Screening for Aryl Hydrocarbon Receptor Agonistic Activity in 200 Pesticides Using a Highly Sensitive Reporter Cell Line, DR‐EcoScreen Cells, and In Vivo Mouse Liver Cytochrome P450‐1A Induction by Propanil, Diuron and Linuron,” Chemosphere 74, no. 1 (2008): 155–165, 10.1016/j.chemosphere.2008.08.015. 18835618

[17] H. Kojima , E. Katsura , S. Takeuchi , K. Niiyama , and K. Kobayashi , “Screening for Estrogen and Androgen Receptor Activities in 200 Pesticides by in Vitro Reporter Gene Assays Using Chinese Hamster Ovary Cells,” Environmental Health Perspectives 112, no. 5 (2004): 524–531, 10.1289/ehp.6649. 15064155 PMC1241915

[18] L. A. Konickx , F. Worek , S. Jayamanne , H. Thiermann , N. A. Buckley , and M. Eddleston , “Reactivation of Plasma Butyrylcholinesterase by Pralidoxime Chloride in Patients Poisoned by WHO Class II Toxicity Organophosphorus Insecticides,” Toxicological Sciences 136, no. 2 (2013): 274–283, 10.1093/toxsci/kft217. 24052565 PMC3858199

[19] P. Kokilavani , U. Suriyakalaa , P. Elumalai , et al., “Antioxidant Mediated Ameliorative Steroidogenesis by Commelina benghalensis L. and Cissus quadrangularis L. Against Quinalphos Induced Male Reproductive Toxicity,” Pesticide Biochemistry and Physiology 109 (2014): 18–33, 10.1016/j.pestbp.2014.01.002. 24581381

[20] M. M. Babín and J. V. Tarazona , “In Vitro Toxicity of Selected Pesticides on RTG‐2 and RTL‐W1 Fish Cell Lines,” Environmental Pollution 135, no. 2 (2005): 267–274, 10.1016/j.envpol.2004.11.001. 15734586

[21] V. Mishra and N. Srivastava , “Organophosphate Pesticides‐Induced Changes in the Redox Status of Rat Tissues and Protective Effects of Antioxidant Vitamins,” Environmental Toxicology 30, no. 4 (2015): 472–482, 10.1002/tox.21924. 24248738

[22] G. Wu , E. Dawson , A. Duong , R. Haw , and L. Stein , “ReactomeFIViz: A Cytoscape App for Pathway and Network‐Based Data Analysis,” F1000Research 3 (2014): 146, 10.12688/f1000research.4431.2. 25309732 PMC4184317

[23] J. K. Choudhari , M. K. Verma , J. Choubey , and B. P. Sahariah , “Investigation of MicroRNA and Transcription Factor Mediated Regulatory Network for Silicosis Using Systems Biology Approach,” Scientific Reports 11, no. 1 (2021): 1265, 10.1038/s41598-020-77636-4. 33446673 PMC7809153

[24] J. K. Choudhari , M. K. Verma , and B. P. Sahariah , “Chronic Fatigue Syndrome: Identification of Transcription Factor (TFs) Associated With Gene Expression for Drug Signature Prediction,” Netw Model Anal Health Inform Bioinforma 8, no. 1 (2019): 23, 10.1007/s13721-019-0203-7.

[25] J. K. Choudhari , T. Chatterjee , S. Gupta , J. G. Garcia‐Garcia , and J. Vera‐González , “Network Biology Approaches in Ophthalmological Diseases: A Case Study of Glaucoma,” in Systems Medicine, ed. O. Wolkenhauer (Academic Press, 2021), 190–202.

[26] M. Kuhn , C. von Mering , M. Campillos , L. J. Jensen , and P. Bork , “STITCH: Interaction Networks of Chemicals and Proteins,” Nucleic Acids Research 36, no. Database issue (2008): D684–D688, 10.1093/nar/gkm795. 18084021 PMC2238848

[27] M. Griffith , O. L. Griffith , A. C. Coffman , et al., “DGIdb—Mining the Druggable Genome,” Nature Methods 10, no. 12 (2013): 1209–1210, 10.1038/nmeth.2689. 24122041 PMC3851581

[28] G. D. Bader and C. W. Hogue , “An Automated Method for Finding Molecular Complexes in Large Protein Interaction Networks,” BMC Bioinformatics 4, no. 1 (2003): 2, 10.1186/1471-2105-4-2. 12525261 PMC149346

[29] B. Yashwanth , R. Pamanji , and J. V. Rao , “Toxicomorphomics and Toxicokinetics of Quinalphos on Embryonic Development of Zebrafish (*Danio rerio*) and its Binding Affinity Towards Hatching Enzyme, ZHE1,” Aquatic Toxicology 180 (2016): 155–163, 10.1016/j.aquatox.2016.09.018. 27716580

[30] T. Namba , “Cholinesterase Inhibition by Organophosphorus Compounds and its Clinical Effects,” Bulletin of the World Health Organization 44, no. 1–3 (1971): 289–307, https://pmc.ncbi.nlm.nih.gov/articles/PMC2428032/. 4941660 PMC2428032

[31] M. B. Čolović , D. Z. Krstić , T. D. Lazarević‐Pašti , A. M. Bondžić , and V. M. Vasić , “Acetylcholinesterase Inhibitors: Pharmacology and Toxicology,” Current Neuropharmacology 11, no. 3 (2013): 315–335, 10.2174/1570159x11311030006. 24179466 PMC3648782

[32] O. A. Lenina , I. V. Zueva , V. V. Zobov , V. E. Semenov , P. Masson , and K. A. Petrov , “Slow‐Binding Reversible Inhibitor of Acetylcholinesterase With Long‐Lasting Action for Prophylaxis of Organophosphate Poisoning,” Scientific Reports 10, no. 1 (2020): 16611, 10.1038/s41598-020-73822-6. 33024231 PMC7538863

[33] M. Subramaneyaan , S. Jain , C. Yadav , V. K. Arora , B. D. Banerjee , and R. S. Ahmed , “Quinalphos Induced Oxidative Stress and Histoarcheitectural Alterations in Adult Male Albino Rats,” Environmental Toxicology and Pharmacology 34, no. 3 (2012): 673–678, 10.1016/j.etap.2012.10.003. 23146591

[34] H. Kojima , S. Takeuchi , and T. Nagai , “Endocrine‐Disrupting Potential of Pesticides via Nuclear Receptors and Aryl Hydrocarbon Receptor,” Journal of Health Science 56, no. 4 (2010): 374–386, 10.1248/jhs.56.374.

[35] C. Dietrich and B. Kaina , “The Aryl Hydrocarbon Receptor (AhR) in the Regulation of Cell–Cell Contact and Tumor Growth,” Carcinogenesis 31, no. 8 (2010): 1319–1328, 10.1093/carcin/bgq028. 20106901 PMC6276890

[36] E. Noh , J. M. Moon , B. J. Chun , Y. S. Cho , S. Ryu , and D. Kim , “The Clinical Role of Serum Albumin in Organophospate Poisoning,” Basic and Clinical Pharmacology and Toxicology 128, no. 4 (2021): 605–614, 10.1111/bcpt.13546. 33306270

[37] A. Padmanabha , H. R. V. Reddy , A. Bhat , and M. Khavi , “Quinalphos Induced Oxidative Stress Biomarkers in Liver and Kidney of Common Carp, Cyprinus carpio,” Nature Environment and Pollution Technology 14, no. 4 (2015): 871, https://neptjournal.com/upload‐images/NL‐54‐21‐(19)B‐3140.pdf.

[38] J. T. Busher , “Serum Albumin and Globulin,” in Clinical Methods: The History, Physical, and Laboratory Examinations’, eds. H. K. Walker , W. D. Hall , and J. W. Hurst . 3rd ed. (Butterworths, 1990). 21250045

[39] J. R. Carvalho and M. V. Machado , “New Insights About Albumin and Liver Disease,” Annals of Hepatology 17, no. 4 (2018): 547–560, 10.5604/01.3001.0012.0916. 29893696

[40] K. J. Kinter and A. A. Anekar , Biochemistry, Dihydrotestosterone’ (StatPearls Publishing, 2022). 32491566

[41] Y. H. Jung , C. W. Chae , G. E. Choi , et al., “Cyanidin 3‐O‐arabinoside Suppresses DHT‐Induced Dermal Papilla Cell Senescence by Modulating p38‐Dependent ER‐mitochondria Contacts,” Journal of Biomedical Science 29, no. 1 (2022): 17, 10.1186/s12929-022-00800-7. 35255899 PMC8900350

[42] W. Mnif , A. I. H. Hassine , A. Bouaziz , A. Bartegi , O. Thomas , and B. Roig , “Effect of Endocrine Disruptor Pesticides: A Review,” International Journal of Environmental Research and Public Health 8, no. 6 (2011): 2265–2303, 10.3390/ijerph8062265. 21776230 PMC3138025

[43] N. Pant and S. P. Srivastava , “Testicular and Spermatotoxic Effects of Quinalphos in Rats,” Journal of Applied Toxicology 23, no. 4 (2003): 271–274, 10.1002/jat.919. 12884411

[44] A. Ray , S. Chatterjee , S. Ghosh , K. Bhattacharya , A. Pakrashi , and C. Deb , “Quinalphos‐Induced Suppression of Spermatogenesis, Plasma Gonadotrophins, Testicular Testosterone Production, and Secretion in Adult Rats,” Environmental Research 57, no. 2 (1992): 181–189, 10.1016/s0013-9351(05)80078-7. 1568439

[45] J. Blanco‐Muñoz , M. M. Morales , M. Lacasaña , C. Aguilar‐Garduño , S. Bassol , and M. E. Cebrián , “Exposure to Organophosphate Pesticides and Male Hormone Profile in Floriculturist of the State of Morelos, Mexico,” Human Reproduction 25, no. 7 (2010): 1787–1795, 10.1093/humrep/deq082. 20435691

[46] R. K. Gupta and R. C. Gupta , “Chapter 68—Placental Toxicity,” in Reproductive and Developmental Toxicology, ed. R. C. Gupta . 2nd ed. (Academic Press, 2017), 1301–1325.

[47] S. Darvesh , D. A. Hopkins , and C. Geula , “Neurobiology of Butyrylcholinesterase,” Nature Reviews Neuroscience 4, no. 2 (2003): 131–138, 10.1038/nrn1035. 12563284

[48] N. H. Greig , D. K. Lahiri , and K. Sambamurti , “Butyrylcholinesterase: An Important New Target in Alzheimer’s Disease Therapy,” supplement, International Psychogeriatrics 14, no. S1 (2002): 77–91, 10.1017/s1041610203008676. 12636181

[49] M. Jokanović , “Studies on the Delayed Neuropathic and Anticholinesterase Potential of Quinalphos (Diethyl 2‐Quinoxalyl Phosphorothionate) in Hens,” Journal of Applied Toxicology 13, no. 5 (1993): 337–339, 10.1002/jat.2550130507. 8258630

[50] S. Kohno , M. C. Bernhard , Y. Katsu , et al., “Estrogen Receptor 1 (ESR1; ERα), not ESR2 (ERβ), Modulates Estrogen‐Induced Sex Reversal in the American Alligator, a Species With Temperature‐Dependent Sex Determination,” Endocrinology 156, no. 5 (2015): 1887–1899, 10.1210/en.2014-1852. 25714813 PMC5393338

[51] S. Kitamura , K. Sugihara , and N. Fujimoto , “CHAPTER 34 – Endocrine Disruption by Organophosphate and Carbamate Pesticides,” in Toxicology of Organophosphate & Carbamate Compounds, ed. R. C. Gupta (Academic Press, 2006), 481–494.

[52] S. Gómez‐Manzo , J. Marcial‐Quino , A. Vanoye‐Carlo , et al., “Glucose‐6‐Phosphate Dehydrogenase: Update and Analysis of New Mutations Around the World,” International Journal of Molecular Sciences 17, no. 12 (2016): 2069, 10.3390/ijms17122069. 27941691 PMC5187869

[53] S. Sudsumrit , K. Chamchoy , D. Songdej , et al., “Genotype‐Phenotype Association and Biochemical Analyses of Glucose‐6‐Phosphate Dehydrogenase Variants: Implications for the Hemolytic Risk of Using 8‐Aminoquinolines for Radical Cure,” Frontiers in Pharmacology 13 (2022): 1032938, 10.3389/fphar.2022.1032938. 36339627 PMC9631214

[54] P. Arese , V. Gallo , A. Pantaleo , and F. Turrini , “Life and Death of Glucose‐6‐Phosphate Dehydrogenase (G6PD) Deficient Erythrocytes – Role of Redox Stress and Band 3 Modifications,” Transfusion Medicine and Hemotherapy 39, no. 5 (2012): 328–334, 10.1159/000343123. 23801924 PMC3678266

[55] V. E. Sobolev , M. O. Sokolova , R. O. Jenkins , and N. V. Goncharov , “Molecular Mechanisms of Acute Organophosphate Nephrotoxicity,” International Journal of Molecular Sciences 23, no. 16 (2022): 8855, 10.3390/ijms23168855. 36012118 PMC9407954

[56] A. F. Castillo , J. Fan , V. Papadopoulos , and E. J. Podestá , “Hormone‐Dependent Expression of a Steroidogenic Acute Regulatory Protein Natural Antisense Transcript in MA‐10 Mouse Tumor Leydig Cells,” PLoS One 6, no. 8 (2011): e22822, 10.1371/journal.pone.0022822. 21829656 PMC3148237

[57] J. Lee , T. Tong , H. Duan , et al., “Regulation of StAR by the N‐terminal Domain and Coinduction of SIK1 and TIS11b/Znf36l1 in Single Cells,” Frontiers in Endocrinology 7 (2016), 10.3389/fendo.2016.00107. PMC4969582 27531991

[58] H. Kojima , F. Sata , S. Takeuchi , T. Sueyoshi , and T. Nagai , “Comparative Study of Human and Mouse Pregnane X Receptor Agonistic Activity in 200 Pesticides Using In Vitro Reporter Gene Assays,” Toxicology 280, no. 3 (2011): 77–87, 10.1016/j.tox.2010.11.008. 21115097

[59] D. Debnath and T. K. Mandal , “Study of Quinalphos (An Environmental Oestrogenic Insecticide) Formulation (Ekalux 25 E.C.)‐Induced Damage of the Testicular Tissues and Antioxidant Defence Systems in Sprague‐Dawley Albino Rats,” Journal of Applied Toxicology 20, no. 3 (2000): 197–204, 10.1002/(sici)1099-1263(200005/06)20:3<197::aid-jat634>3.0.co;2-7. 10797472

[60] N. G. Girer , D. Carter , N. Bhattarai , et al., “Inducible Loss of the Aryl Hydrocarbon Receptor Activates Perigonadal White Fat Respiration and Brown Fat Thermogenesis via Fibroblast Growth Factor 21,” International Journal of Molecular Sciences 20, no. 4 (2019): 950, 10.3390/ijms20040950. 30813227 PMC6412252

[61] E. N. Jackson , S. E. Thatcher , N. Larian , et al., “Effects of Aryl Hydrocarbon Receptor Deficiency on PCB‐77‐Induced Impairment of Glucose Homeostasis During Weight Loss in Male and Female Obese Mice,” Environmental Health Perspectives 127, no. 7 (2019): 77004, 10.1289/ehp4133. 31306034 PMC6794491

[62] V. Turcot , Y. Lu , H. M. Highland , et al., “Protein‐Altering Variants Associated With Body Mass Index Implicate Pathways That Control Energy Intake and Expenditure in Obesity,” Nature Genetics 50, no. 1 (2018): 26–41, 10.1038/s41588-017-0011-x. 29273807 PMC5945951

[63] F. O’mahony , M. Razandi , A. Pedram , B. J. Harvey , and E. R. Levin , “Estrogen Modulates Metabolic Pathway Adaptation to Available Glucose in Breast Cancer Cells,” Molecular Endocrinology 26, no. 12 (2058): 17–2070, 10.1210/me.2012-1191. PMC3517720 23028062

[64] R. P. A. Barros and J.‐Å. Gustafsson , “Estrogen Receptors and the Metabolic Network,” Cell Metabolism 14, no. 3 (2011): 289–299, 10.1016/j.cmet.2011.08.005. 21907136

[65] M. Anbalagan , B. Huderson , L. Murphy , and B. G. Rowan , “Post‐Translational Modifications of Nuclear Receptors and Human Disease,” Nuclear Receptor Signaling 10 (2012), 10.1621/nrs.10001. PMC3309075 22438791

[66] R. A. Stein , C. Chang , D. A. Kazmin , et al., “Estrogen‐Related Receptor Alpha is Critical for the Growth of Estrogen Receptor‐Negative Breast Cancer,” Cancer Research 68, no. 21 (2008): 8805–8812, 10.1158/0008-5472.can-08-1594. 18974123 PMC2633645

[67] C. Rachez , B. D. Lemon , Z. Suldan , et al., “Ligand‐Dependent Transcription Activation by Nuclear Receptors Requires the DRIP Complex,” Nature 398, no. 6730 (1999): 824–828, 10.1038/19783. 10235266

[68] Z. Al Tanoury , A. Piskunov , and C. Rochette‐Egly , “Vitamin A and Retinoid Signaling: Genomic and Nongenomic Effects: Thematic Review Series: Fat‐Soluble Vitamins: Vitamin A,” Journal of Lipid Research 54, no. 7 (2013): 1761, 10.1194/jlr.R030833. 23440512 PMC3679380

[69] Q. Wang , W. J. Zhang , L. Y. He , and X. F. Sheng , “Increased Biomass and Quality and Reduced Heavy Metal Accumulation of Edible Tissues of Vegetables in the Presence of Cd‐Tolerant and Immobilizing Bacillus megaterium H3,” Ecotoxicology and Environmental Safety 148 (2018): 269–274, 10.1016/j.ecoenv.2017.10.036. 29069614

[70] S. Karami , G. Andreotti , S. Koutros , et al., “Pesticide Exposure and Inherited Variants in Vitamin D Pathway Genes in Relation to Prostate Cancer,” Cancer Epidemiology Biomarkers & Prevention: A publication of the American Association for Cancer Research, cosponsored by the American Society of Preventive Oncology 22, no. 9 (2013): 1557–1566, 10.1158/1055-9965.EPI-12-1454. PMC3773544 23833127

[71] X. Wang , M. Li , X. Zhang , et al., “CYP11A1 Upregulation Leads to Trophoblast Oxidative Stress and Fetal Neurodevelopmental Toxicity That can be Rescued by Vitamin D,” Frontiers in Molecular Biosciences 7 (2021), 10.3389/fmolb.2020.608447. PMC7917044 33659272

[72] L. Xiao , Z. Yu , H. Liu , et al., “Effects of Cd and Pb on Diversity of Microbial Community and Enzyme Activity in Soil,” Ecotoxicology (London, England) 29, no. 5 (2020): 551–558, 10.1007/s10646-020-02205-4. 32394358

[73] A. B. Shintyapina , V. A. Vavilin , O. G. Safronova , and V. V. Lyakhovich , “The Gene Expression Profile of a Drug Metabolism System and Signal Transduction Pathways in the Liver of Mice Treated With Tert‐Butylhydroquinone or 3‐(3’‐Tert‐Butyl‐4’‐Hydroxyphenyl)Propylthiosulfonate of Sodium,” PLoS One 12, no. 5 (2017): e0176939, 10.1371/journal.pone.0176939. 28467491 PMC5415222

[74] M. Kanehisa and S. Goto , “KEGG: Kyoto Encyclopedia of Genes and Genomes,” Nucleic Acids Research 28, no. 1 (2000): 27–30, 10.1093/nar/28.1.27. 10592173 PMC102409

[75] A. Fabregat , K. Sidiropoulos , G. Viteri , et al., “Reactome Pathway Analysis: A High‐Performance in‐Memory Approach,” BMC Bioinformatics 18, no. 1 (2017): 142, 10.1186/s12859-017-1559-2. 28249561 PMC5333408

[76] T. P. Knutson and C. A. Lange , “Dynamic Regulation of Steroid Hormone Receptor Transcriptional Activity by Reversible SUMOylation,” Vitamins and hormones 93 (2013): 227–261, 10.1016/B978-0-12-416673-8.00008-3. 23810010

[77] S. S. Zaidi , V. K. Bhatnagar , S. J. Gandhi , M. P. Shah , P. K. Kulkarni , and H. N. Saiyed , “Assessment of Thyroid Function in Pesticide Formulators,” Human & Experimental Toxicology 19, no. 9 (2000): 497–501, 10.1191/096032700677928536. 11204551

[78] A. T. Slominski , T. K. Kim , W. Li , A. K. Yi , A. Postlethwaite , and R. C. Tuckey , “The Role of CYP11A1 in the Production of Vitamin D Metabolites and Their Role in the Regulation of Epidermal Functions,” Journal of Steroid Biochemistry and Molecular Biology 144, no. (PART A) (2014): 28–39, 10.1016/j.jsbmb.2013.10.012. 24176765 PMC4002668

[79] E. Treuter and N. Venteclef , “Transcriptional Control of Metabolic and Inflammatory Pathways by Nuclear Receptor SUMOylation,” Biochimica et Biophysica Acta 1812, no. 8 (2011): 909–918, 10.1016/j.bbadis.2010.12.008. 21172431

